# Multi-omics Signatures and Translational Potential to Improve Thyroid Cancer Patient Outcome

**DOI:** 10.3390/cancers11121988

**Published:** 2019-12-10

**Authors:** Myriem Boufraqech, Naris Nilubol

**Affiliations:** Surgical Oncology Program, National Cancer Institute, National Institutes of Health, Bethesda, MD 20817, USA; myriem.boufraqech@nih.gov

**Keywords:** genomic, proteomic, methylation, microRNA, thyroid cancer

## Abstract

Recent advances in high-throughput molecular and multi-omics technologies have improved our understanding of the molecular changes associated with thyroid cancer initiation and progression. The translation into clinical use based on molecular profiling of thyroid tumors has allowed a significant improvement in patient risk stratification and in the identification of targeted therapies, and thereby better personalized disease management and outcome. This review compiles the following: (1) the major molecular alterations of the genome, epigenome, transcriptome, proteome, and metabolome found in all subtypes of thyroid cancer, thus demonstrating the complexity of these tumors and (2) the great translational potential of multi-omics studies to improve patient outcome.

## 1. Introduction

The incidence of thyroid cancer has been increasing by almost 300% in the past four decades with an estimated 54,000 patients diagnosed each year in the United States [1]. Thyroid cancer is the fifth most common cancer in women. Earlier data has suggested that the rising incidence of thyroid cancer has been due to the increased detection of small, commonly incidental, and subclinical papillary thyroid cancer (PTC) with indolent behavior [1,2,3,4], however, the more recent analysis of the Surveillance, Epidemiology, and End Results (SEER) cancer registry data has shown an increase in advanced-stage PTC and PTC larger than 5 cm which were believed to be clinically detectable or were symptomatic [5]. From 1994 to 2013, incidence-based mortality from PTC increased 1.1% per year overall and 2.9% per year in those with metastatic PTC, consistent with a true increase in the incidence of thyroid cancer in the United States [6]. Thus, it is critically important to understand the molecular events associated with thyroid cancer initiation and progression to improve diagnostic accuracy, risk stratification, and to personalize treatment and surveillance plans.

Primary thyroid cancers originate from two distinct types of cells, thyroid follicular cells derived from endoderm and parafollicular cells (also known as C cells) derived from the neural crest. The majority of thyroid cancers originate from thyroid follicular cells that give rise to two major histologic subtypes, PTC and follicular thyroid cancer (FTC). Medullary thyroid cancer (MTC) derives from C-cells and has its unique clinical characteristics and more uniform molecular features that have been discussed elsewhere.

## 2. Well-Differentiated Thyroid Cancer

PTC is the most common histologic subtype of thyroid cancer in the United States and other iodine sufficient areas, accounting for more than 80% of thyroid cancers. The classical variant PTC (cPTC) is the most common histologic variant of PTC, accounting for approximately two-thirds of all diagnosed PTC, including papillary thyroid microcarcinoma, defined as PTC smaller than 1 cm. PTC is diagnosed based on the characteristic nuclear features [7]. The presence of true papillae and a fibrovascular core is seen in cPTC, while follicular variant PTC (FVPTC) is diagnosed based on the follicular pattern and the absence of papillae. FVPTC can be further subclassified by the presence and the degree of invasion [7,8]. Encapsulated FVPTC has been reclassified and renamed as noninvasive follicular thyroid neoplasm with papillary-like nuclear features (NIFT-P) because of the low malignant potential and benign clinical course. Another histologic variant of PTC includes tall cell variant PTC (TVPTC) which is characterized by a height greater than twice its width with abundant eosinophilic cytoplasm and basilar oriented nuclei. TVPTC has a more aggressive behavior with high rates of local, regional, distant metastasis, and locoregional recurrence [7,9]. Rare histologic variants of PTC include solid, diffuse sclerosing, columnar, trabecular, oncocytic, and hobnail. These variants are associated with high rates of metastasis and thyroid cancer-related mortality [10,11], and thus are sometimes categorized as poorly differentiated thyroid cancer (PDTC). The cribriform-morular variant PTC is a characteristic variant associated with familial adenomatous polyposis (FAP) [12].

FTC is a less common type of thyroid cancer, particularly in iodine sufficient countries, accounting for 5% to 10% of thyroid cancer. The decrease in diagnosis of a true FTC can be from the increase in the iodine-supplemented salt and the changes in the diagnostic pattern of FVPTC that may have been misdiagnosed in the past as FTC [13,14]. The pathological spectrum of follicular neoplasms includes follicular adenoma, minimally invasive FTC, widely invasive FTC [15], and arguably Hurthle cell adenoma and Hurthle cell carcinoma (HCC). As compared with follicular adenoma, the diagnosis of FTC is established based on the presence of capsular or vascular invasion. Although the overall prognosis is excellent, similar to that of cPTC, FTC has a lower rate of lymph node metastasis and a higher rate of hematogenous metastasis [13]. Similar to FTC, the diagnosis of HCC is established based on capsular or vascular invasion, and also similar to FTC, HCC metastasizes to distant sites via hematogenous spread. However, unlike FTC, HCC has higher rates of lymph node metastasis and local/regional recurrence with a lower rate of iodine uptake ability [16]. However, data from the National Cancer Database has suggested that radioactive iodine improved survival in patients with HCC greater than 2 cm, and in patients with nodal and distant metastasis [17].

## 3. Poorly Differentiated Thyroid Cancer (PDTC)

Although the precise criteria to diagnose PDTC is debatable, the diagnosis of PDTC is made based on the reduced thyroid differentiation and a presence of nuclear pleomorphism on histology. Common histologic features include increased cell proliferative indices, capsular or vascular invasion, and focal tumor necrosis. Insular thyroid cancer is distinctly recognized as PDTC initially and the classification has expanded to include some of the high-grade PTC variants mentioned above. These tumors are often radioactive iodine resistant and are associated with high rates of metastasis and thyroid cancer-related deaths.

## 4. Anaplastic Thyroid Cancer (ATC)

ATC is one of the most aggressive cancers in humans with rapid tumor growth, and high rates of local recurrence, distant metastasis, and mortality. The median survival of patients with ATC is 5 months. Fortunately, ATC accounts for only 1.7% of all thyroid cancers [18]. A multidisciplinary team comprised of surgeons, radiologists, medical and radiation oncologists, palliative care physicians, and endocrinologists is required to establish the optimal treatment strategy for patients. Recently, insight into the molecular pathology of ATC has led to the FDA-approved combination treatment of dabrafenib (BRAF inhibitor) and trametinib (MEK inhibitor) in BRAF-mutated ATC. This combination treatment has resulted in an unprecedented response rate of 69% and a twelve-month progression-free survival of 79% [19].

## 5. Genomics of Thyroid Cancer

The rapid improvement of high-throughput, multi-omic assays and the collaborative efforts to systemically use multiplatform analyses to advance knowledge in the pathophysiology of cancer initiation and progression has produced a wealth of data which is now available publicly. These include the data from the Cancer Genome Atlas, the International Cancer Genome Consortium, the National Center for Biotechnology Information, and several other online resources. Specific genomic alterations by thyroid cancer histology are discussed below.

## 6. Papillary Thyroid Carcinoma (PTC)

**Figure 1:**
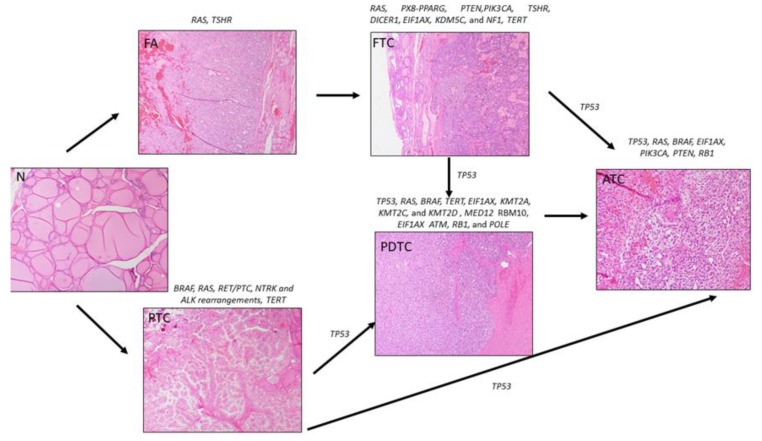
Genetic alterations involved in thyroid cancer initiation or progression. Abbreviations: N = normal thyroid, FA = follicular adenoma, FTC = follicular thyroid cancer, PTC = papillary thyroid cancer, PDTC = poorly-differentiated thyroid cancer, and ATC = anaplastic thyroid cancer.

As compared with other cancers in the TCGA database, PTC is a tumor with one of the most stable genomes because it is one of lowest mutational burdens (0.41 nonsynonymous somatic mutations per megabase of DNA) with few copy number alterations. The indolent clinical course of PTC is likely related to a relatively stable genome and low mutation burden. Thyroid cancer samples in TCGA were mostly low risk cPTCs that usually carry only a single gene mutation or gene fusion. Given the rapid expansion of massively parallel sequencing technology, the knowledge of molecular pathology in thyroid cancer has grown. The TCGA database identified the following two major gene expression signatures in thyroid cancers (mostly PTC): *BRAF* P.V600E-like and *RAS-*like groups. These mutations and gene fusions activate the receptor tyrosine kinase (RTK)/mitogen-activated protein kinase (MAPK) signaling pathway that regulates thyroid cell proliferation, differentiation, cell-cell adhesion, migration, and cell survival (Figure 1). These mutations and gene fusions are almost always mutually exclusive [20].

### 6.1. BRAF Mutations

*BRAF* is the most common somatic mutations in PTC, accounting for 40% to 65% of all PTCs [21]. Greater than 95% of *BRAF* mutations in PTC is p.V600E [22]. Other *BRAF* mutations, such as p. K601E, are associated with lower oncogenic potential [23,24]. *BRAF* is an oncogene that encodes a serine-threonine protein kinase which is the key regulator of the MAPK pathway. *BRAF* p.V600E is associated with cPTC and TVPTC with a strong MAPK signaling and reduced follicular cell differentiation and lower iodine uptake and metabolism [21,25]. The data from TCGA showed that BRAF-like and RAS-like mutations are mutually exclusive. Thus, *BRAF* p.V600E is less common in NIFT-P and FVPTC. As compared to cPTC, the rate of *BRAF* p.V600E mutation in TVPTC is higher, ranging from 80% to 100% [26].

The clinical utility of *BRAF* p.V600E is to improve diagnostic accuracy of fine-needle aspiration biopsy of indeterminate thyroid nodules, as the presence of *BRAF* p.V600E in the aspirate is almost synonymous with PTC with a high positive predictive value (95% to 100%). However, because of the poor sensitivity (~50% in cytologically suspicious in PTC), it remains unclear if the use of *BRAF* p.V600E as a single molecular testing is cost-efficient [27,28]. The prognostic value of *BRAF* p.V600E mutated thyroid cancer is still the subject of controversy. A meta-analysis of 27 studies (n = 5655) suggests the association between *BRAF* p.V600E mutated thyroid cancer and extrathyroidal extension, lymph node metastasis, more advanced stage [29]. Patients with solitary intrathyroidal *BRAF* p.V600E mutated thyroid cancer are at a higher risk for recurrence [30]. Although *BRAF* p.V600E mutation in patients with PTC is associated with poor prognostic features, *BRAF* p.V600E mutation is not an independent prognostic factor for PTC-related mortality [31]. Subsequent studies have been conducted to identify a subset of patients with PTC-related mortality associated with *BRAF* p.V600E mutation. Unlike patients with wild type *BRAF* thyroid cancer, a linear association between thyroid cancer mortality and age in patients with *BRAF* p.V600E mutations has been observed and has been found to be independent to other clinicopathologic risk factors [32]. Male sex is also an independent risk factor for PTC-specific mortality in patients with *BRAF* p.V600E, but not in those with wild type *BRAF* [33].

### 6.2. RAS Mutations

*RAS* genes (*NRAS, HRAS,* and *KRAS*) are the most common oncogenes in human cancers with high rates of somatic mutations in pancreatic, lung, and colorectal cancers. A member of the *RAS* family genes encodes a class of proteins, called small GTPase, that regulates intracellular signaling transduction that activates the MAPK pathway affecting cell growth, differentiation, and cell survival. The missense mutations affect the GTP-binding domain at exon 2 (codons 12 and 13) and at exon 3 (codon 61) result in a constitutive activation of the MAPK signaling pathway as the protein is locked in a GTP-bound form [34,35]. A high prevalence of *RAS* mutations in PTC is commonly observed in FVPTC and NIFT-P (30% to 50%) [21] but not in cPTC. The frequency of *NRAS, HRAS,* and *KRAS* in cPTC from the TCGA database was 4%, 1.5%, and 0.3%, respectively [22]. Of note, *RAS* mutations are frequently observed in benign lesions such as follicular adenoma, as well as PDTC and ATC (see below).

### 6.3. Other Mutations

Similar to the TCGA cohort, the PTC cohort from China had a high prevalence of *BRAF* p.V600E mutation (59%). However, the second most frequently altered gene was the long non-coding RNA called *GAS8-AS1* (9.2%) which has tumor-suppressive functions, followed by *RAS* (3.2%) and a novel mutation in the *LPAR4* gene (2.7%) [36]. A genomic study performed in a large cohort of PTC from Saudi Arabia (n = 886), where thyroid cancer is the second most common cancer in women, showed a high prevalence of *BRAF (*59%)*, HRAS* (2%), and *NRAS* (1%)*,* similar to other cohorts. However, the third most frequently altered gene was *TG* (3%) encoding thyroglobulin. Patients with *TG* alternation had significantly higher rates of disease recurrence and metastasis, however, 78% of the patients with *TG* alternation had coexisting mutations in the MAPK pathway suggesting that *TG* alteration may be associated with tumor progression [37].

Mutations in the phosphoinositide 3-kinase (PI3K) pathway (PI3K/PTEN/AKT/mTOR) have been reported at low frequencies [31]. Mutations in the WNT signaling pathway have been found in only 1.5% of thyroid cancer in the TCGA cohort [22].

## 7. Gene Rearrangements

### 7.1. RET/PTC Rearrangements

The most common gene arrangements in PTC involve *RET* oncogene representing 8% and 4% of mutations in cPTC and FVPTC in the TCGA database, respectively [22]. There have been at least 20 different *RET* rearrangements such as RET/PTC fusion proteins 1 to 9 [38]. The RET/PTC1 (CCDC6-RET) is the most common rearrangement, accounting for 60% of thyroid cancer with *RET* rearrangements, followed by RET/PTC3 (NCOA4-RET, 30%), and RET/PTC2 (PRKAR1A-RET, 5%). These RET/PTC rearrangements are specifically identified in PTC [25]. These fusions result in the constitutive activation of the intracellular domain of the receptor and the uncontrolled MAPK and PI3K signaling pathways [38].

RET/PTC rearrangements have been strongly associated with radiation exposure. Unlike *RET/PTC1* rearrangement that is common in PTC and is not commonly associated with radiation exposure, *RET/PTC3* rearrangement is commonly seen following radiation exposure and is associated with solid variants of PTC and a more aggressive clinical course [39,40]. The prevalence of RET/PTC rearrangements has been up to 87% from the initial series reported after the Chernobyl disaster [41]. Approximately 20% of the pediatric PTCs had RET/PTC1 or RET/PTC3 rearrangement [42]. RET/PTC rearrangements have been reported in benign thyroid diseases such as a high prevalence of RET/PTC found in patients with Hashimoto thyroiditis [43].

### 7.2. NTRK Rearrangements

*NTRK1, 2,* and *3* encode the neurotrophin receptor TRKA, B, and C, respectively. The binding to the extracellular regions of the TRK proteins results in the activation of intracellular tyrosine kinase domains and activation of downstream MAPK, PI3K, and PKC signaling pathways [44]. The *NTRK* fusion-positive tumors, other than PTC, include colorectal cancer, lung cancers (large cell neuroendocrine carcinoma and NSCLC), infantile fibrosarcoma, and pilocytic astrocytoma [44]. The *NTRK* rearrangements found in PTC includes *NTRK1* with *TPM3, TPR*, and *TFG*, and *NTRK3* with *ETV6*. ETV6/NTRK3 rearrangements have been reported as the second most common oncogenic rearrangements in radiation-associated PTCs (after RET/PTCs) accounting for 14.5% of such tumors [45]. TPM3-NTRK1 and TPR-NTRK1 have been identified in radiation-associated PTCs.

In pediatric PTC in the US, a high prevalence of NTRK rearrangements (most commonly ETV6/NTRK3) have been identified [42]. The features commonly found in pediatric PTC with ETV6/NTRK rearrangement have included nodular and infiltrative architecture and lymphovascular invasion.

Although NTRK rearrangements in thyroid cancer do not frequently occur, there are TRK inhibitors such as entrectnib and larotrectinib available in clinical trials for advanced solid cancers with NTRK rearrangements that may benefit patients with advanced NTRK-rearranged PTCs [46].

### 7.3. ALK Rearrangments

The prevalence of *ALK* rearrangements in PTC is ~1% to 5% (1.23% in the TCGA database). Most common *ALK* rearrangements are STRN/ALK, TFG/ALK, and EML4/ALK. *EML4-ALK* rearrangement is typically seen in NSCLC as well. The fusions result in an increased *ALK* mRNA expression, more than 30-fold, leading to constitutive MAPK activation. *STRN-ALK* rearrangement results in increased TSH-independent cell proliferation and fibrosarcoma-like transformation. PTCs with *ALK* rearrangements predominantly have a follicular growth pattern and areas of papillae formation [47]. The associations between *ALK* rearrangement and the tumor aggressiveness and dedifferentiation remain controversial. The initial study demonstrated the association between *ALK-*fusions and follicular growth pattern (seen in FVPTC) extrathyroidal extension, lymph node, and distant metastasis [47]. However, a large cohort of 259 thyroid cancers only found *ALK* rearrangements to be more common in young females and diffuse sclerosing variant PTC, but *ALK-*fusion positive PTCs do not appear to behave more aggressively [48].

Because *ALK* inhibitors such as crizotinib, ceritinib, and alectinib are currently available and FDA approved for *ALK-*fusion positive NSCLC, patients with advanced *ALK* fusion-positive thyroid cancer may respond to *ALK* inhibitors.

Other gene rearrangements in PTC identified in the TCGA database included *BRAF* (2.47%), *PAX8-PPARG* (0.93%), *THADA* (0.31%), *FGFR2* (0.31%), and *LTK* (0.31%). These alterations were also mutually exclusive with each other.

Diffusing sclerosing variant is an aggressive histologic subtype of PTC. Similar to PTC, all mutations and rearrangements are mutually exclusive. In a cohort from Korea, *RET/PTC1* rearrangement (46%) was the most common genetic alteration, followed by *BRAF* p.V600E (24%), and *RET/PTC3* rearrangement (16%). Patients with *RET/PTC3* rearrangement in their tumors had higher rates of T4 stage, distant metastasis, and persistent disease [49].

The overexpression of NF-κB transcription factors is common in thyroid cancer, particularly in PDTC and ATC, and is associated with larger tumor size, nodal metastases, extrathyroidal extension, and *BRAF* p.V600E mutation [50,51,52]. NF-kb is a ubiquitous transcription factor that regulates cell survival, proliferation, and differentiation as well as cellular and immune responses to cytokines and oxidative stress. The role of NF-κB signaling in thyroid cancer cell growth, migration, invasion, and angiogenesis has been shown [53,54,55]. The induction of *RET/PTC1* and *BRAF* p.V600E in thyrocytes and thyroid cancer cells resulted in the activation of NF-kB [56,57]. The NF-kB activation increased anti-apoptotic proteins and cell invasion via increased matrix metalloproteinases. In addition, inactivation of *PTEN* and *PPARG* increased thyroid cancer aggressiveness and was associated with increased NF-kB activation [58,59]. The role of NF-kB in the regulation of thyroid-specific genes such as *TPO, NIS, TG, PAX8,* and *TTF1* has been demonstrated in a mouse model with a thyroid-specific knock-out of the NF-kB essential modulator gene. The mice developed thyroid hypoplastic thyroid and hypothyroidism with a reduction in these thyroid-specific proteins [60,61]. The treatment of PDTC and ATC cells with small molecule inhibitors targeting NF-kB signaling pathway was shown to be effective in the reduction of cell growth and invasion [62,63].

## 8. Tumor Microenvironment in Thyroid Cancer

The tumor microenvironment plays a fundamental role in thyroid cancer progression and metastasis. It includes fibroblast, immune cells, chemokines, cytokines, and growth factors. A growing body of evidence has demonstrated the role of chemokines in modulating the tumor microenvironment and in helping cancer cells evasion from the immune response. These pro-tumorigenic secreted factors include TGFβ, CCL15, CXCL12, CXCL16, and CXCL8. Recent studies have demonstrated an association between the immune microenvironment and mutation status, histological type, and tumor aggressiveness [64,65,66]. Hypoxia, commonly seen in central part of solid cancers, is another key regulator of the tumor microenvironment. An hypoxic environment leads to the secretion of TGF-β, bFGF, and PDGF-β that stimulate the transformation of fibroblasts into cancer-associated fibroblasts (CAFs) [67,68,69]. CAFs are involved in cancer initiation and progression by modulating the ECM, promoting angiogenesis, and stimulating macrophage infiltration [70,71]. *BRAF* p.V600E mutated PTC showed a higher expression of CAFs-associated proteins, suggesting that the aggressiveness of some BRAF mutated tumors might be associated with CAFs [72].

## 9. Follicular Thyroid Cancer (FTC)

Similar to PTC, FTC is a well-differentiated thyroid cancer arising from thyroid follicular cells with the ability to concentrate iodine. However, FTC is distinctly different from PTC in the genetic and molecular features that give rise to its clinical characteristics and pathologic features. FTC represents 5% to 10% of all thyroid cancers in iodine sufficient countries. The prevalence of FTC in some iodine-deficient areas is close to that of PTC [73].

The genetic alteration in FTC (and follicular adenoma) includes predominant *RAS* point mutations, representing 38% to 50%. The most common genetic alteration in FTC is *NRAS* mutations in codon 61 [25] and it has been significantly associated with distant metastasis [74]. The prevalence of RAS codon 61 mutation in follicular adenoma and in FTC are approximately five times higher in an iodine-deficient area [75]. The *PAX8-PPARG* rearrangement is the second most common genetic alteration in FTC. The rate of *PAX8-PPARG* gene fusion ranges from 20% to 50% of FTC, in a smaller percentage of FVPTC, and rarely in FTA [76,77,78].

*PAX8* encodes a paired-box transcription factor that is highly expressed in thyroid follicular cells and is required for the formation of follicular cells [79,80]. Because another rare gene arrangement in FTC, *CREB3L2-PPARG,* has been reported in up to 3% of FTC, [81] this suggests that the PPARG segment of the fusion gene is oncogenesis as these fusions induce high levels of PPRAG expression. However, the mechanisms involved in tumorigenesis and progression remains unclear. Unlike other driver mutations in thyroid cancers that overexpress kinase signaling pathways, *PAX8-PPARG* gene fusion involves tumor immunology and several cancer-related pathways such as apoptosis, cell cycle, and motility [82].

Patients with FTCs harboring the *PAX8-PPARG* gene fusion are typically younger and vascular invasion is frequently observed. The solid or nested area on histology is more common than the wild type tumors. FTC with *PAX8-PPARG* gene fusion tends to pursue a relatively indolent clinical course [76,83]. Other known dysregulated pathways and genes involved in FTC include the PI3K/PTEN/AKT signaling pathway [84].

Somatic mutations of the phosphate and tensin homolog deleted on chromosome ten (*PTEN*) tumor suppressor gene occur in up to 7% to 10% of sporadic FTCs [85,86]. Cowden syndrome is an autosomal dominant disease that is commonly associated with germline *PTEN* mutations resulting in benign and malignant neoplasms and hamartomas. Thyroid pathology associated with Cowden syndrome includes multinodular hyperplasia, follicular adenoma, PTC, and FTC. Activating mutations of PIK3CA also occur in up to 10% of FTCs [87] (Figure 1).

*TERT* promoter mutations and *TP53* mutations can be found in a subset of FTCs, where they have been associated with aggressive disease and poor outcome [88,89]. Recent genomic studies in FTC have shown additional recurrent mutations in *TSHR, DICER1, EIF1AX, KDM5C,* and *NF1.* Nonsynonymous mutation burden in FTC has been associated with disease-specific survival [88].

## 10. Hurthle Cell (Oncocytic) Thyroid Neoplasms

Hurthle cell thyroid carcinoma (HTC) accounts for 3% of all thyroid cancers [90]. These tumors are characterized by their unique histologic findings of oncocytic or oxyphilic cells that are large with increased granular cytoplasm, large nuclei, and an increased cytoplasmic-to-nuclear ratio. The cells have an increased number of abnormally enlarged mitochondria. Similar to the diagnosis of FTC, the diagnostic criteria for HTC, compared to Hurthle cell adenoma, include capsular and/or vascular invasion or presence of lymph node or distant metastasis. Although HTC is similar to FTC that both feature vascular invasion and distant metastasis, HTC is distinctly different than FTC as it has a higher rate of lymph node metastasis and locoregional recurrence and a much lower rate of radioiodine avidity. However, HTC commonly exhibits 18F-FDG avidity [13]. Thus, HTC is now recognized by the WHO to be a distinct entity from FTC [15]. The evidence from a comprehensive integrated high-throughput analysis of mutations, gene expression profiling, and copy number alterations in HCC showed a distinct profile from those of PTC and FTC [91]. Unlike FTC and PTC, HTC had lower rates of RAS mutation and PAX8–PPARG rearrangement and had no *BRAF* or *RET/PTC* rearrangement, respectively [76,91]. Widely invasive HTC, defined as HTC that are grossly invasive, had an extrathyroidal and/or vascular invasion or encapsulated HTC with 4 or more foci of vascular invasion, had differentially expressed genes strongly enriched in PI3K/PTEN/AKT signaling and WNT/b-catenin pathway. In addition, *TP53,* nuclear mitochondrial complex 1, and *PTEN* mutations have been reported in HCC without features of poorly differentiated features [92,93]. The disruption of mitochondrial DNA mutations in complex 1 subunit and mutations in the genes involved in the activators of mitochondrial biogenesis have been reported. These alterations may be responsible for the dysregulated mitochondrial copy number seen in HTC [94,95].

## 11. Poorly Differentiated Thyroid Cancer (PDTC)

Although PDTC accounts for less than 10% of thyroid cancers, the mortality associated with PDTC remains high with a median survival of 3 years. Almost all PDTCs are iodine-refractory. To effectively treat patients with PDTC, it is critical to understand the molecular alterations in PDTC. In contrast to differentiated thyroid cancer, PDTC has additional somatic mutations with the increased mutational burden. The study using the next-generation sequencing platform for targeted sequencing of selected 341 genes (MSK-IMPACT) in 84 samples of PDTC showed somatic mutations commonly seen in differentiated thyroid cancer such as *BRAF* p.V600E (33%) and *RAS* (28%). Other recurrent somatic mutations in PDTC included *TERT* (40%), *EIF1AX* (11%), *TP53* (8%), histone methyltransferase (7%) such as *KMT2A, KMT2C,* and *KMT2D,* mismatch repair genes (2%) including *MSH2 MSH6*, and *MLH1.* Additional mutations include *ARID1B*, *PIK3CA*, and *PTEN* [96].

*EIF1AX* mutations were associated with larger tumors and predicted shorter survival in PDTCs. *TERT* promoter mutations were associated with aggressive behavior and metastasis. Unlike other driver mutations in thyroid cancers, *TERT* promoter mutations can co-occur in tumors with *BRAF* or *RAS* mutations in PDTC and ATC (see below). Distant metastases were more common in *TERT*-mutated PDTCs (56% vs. 20%, *p* = 0.01).

Genomic studies in fatal non-anaplastic thyroid cancer showed a high prevalence of *TERT* promoter mutations, comparable to that of ATC with co-occurrence with *BRAF* or *RAS* mutations. Novel genes altered in fatal non-anaplastic thyroid cancer include *MED12* mutations (14%) and *RBM10* (11%). *MED12* mutations were mutually exclusive to cancers with *TERT* promoter mutations and *BRAF* p.V600E. *RBM10* was found to co-occur with *NRAS.* Other common mutations in fatal non-anaplastic thyroid cancer include mutations in genes involved in *PI3K/AKT/PTEN/mTOR* pathway, *TP53, ATM, RB1,* and *POLE* as well as genes involved in the chromatin remodeling complex and histone methyltransferases [97] (Figure 1) and 11% of PDTCs harbored *EIF1AX* mutations which were strongly associated with *RAS.*

Chromosomal copy number alterations occurring in PDTC mostly are a tumor type– and gene context-specific fashion. In PDTC without known driver mutations, chromosome 1p, 13q, and 15q losses were enriched whereas loss of 22q was strongly associated with *RAS-*mutated PDTCs. Patients with PDTCs with chromosome 1q gains had worse survival [96].

## 12. Anaplastic Thyroid Cancer

ATC is one of the most lethal cancers in humans. It is uniformly invasive with high rates of metastasis and tumor growth regardless of histologic types (spindle cell, squamous, or epithelioid). ATC lacks most markers seen in thyroid cancer such as thyroglobulin, TTF1, NIS, and TSHR, except PAX8 that can be present consistently in squamous variant ATC but in only 50% of ATC with spindle cell features [98]. The co-occurrence with other well or poorly differentiated thyroid cancers suggests the dedifferentiation process from the original tumors [99]. Unlike differentiated thyroid cancers, ATC harbors several additional changes that increase genomic instability such as aneuploidy, copy number alterations, and higher mutation burden that often coexists with known driver mutations in differentiated thyroid cancers [96,100,101]. As compared to PDTCs, ATCs had a higher frequency of mutations in *TP53* (73%), *TERT* promoter (73%), PI3K/AKT/mTOR pathway effectors (39%), SWI/SNF subunits (36%), and histone methyltransferases (24%) and 9% of ATCs had *EIF1AX* mutations. However, no gene rearrangements were observed in the MSKCC series. In ATC genomes, 8p and 17p chromosomal losses and 20q gains were far more frequent as compared with PDTC (Figure 1). In ATCs, 13q losses and 20q gains were associated with shorter survival [96].

## 13. Familial Non-Medullary Thyroid Cancer

Approximately 3% to 9% of non-medullary thyroid cancer have an inheritable pattern of disease. Known familial tumor syndromes that feature thyroid cancer account for only 5% of all familial non-medullary thyroid cancer (FNMTC). These syndromes include familial adenomatous polyposis (Gardner’s syndrome), Cowden syndrome, Werner syndrome, Carney complex, and DICER1 mutation syndrome [102]. Much effort to identify susceptibility genes involved in non-syndromic familial thyroid cancer has resulted in the identification of *FOXE1, HABP2,* and *TITF1. FOXE1* is a transcription factor that regulates thyroglobulin and thyroperoxidase gene expression. The association was initially found with sporadic PTC [103,104] and, subsequently, *FOXE1* germline mutation has been reported in a Portuguese cohort with FNMTC [105]. Mutant variant studied in rat normal thyroid cells and human PTC cell lines showed increased cell proliferation and migration. However, *FOXE1* gene penetrant appears to be low for FNMTC. *HAPB2* encodes hyaluronan-binding protein 2 and has been identified in seven affected members of an FNMTC kindred with PTC and follicular adenoma. Functional studies have shown the mutant variant increased tumor colony formation and cell migration consistent with the loss of tumor suppressor effect. The *HABP2* G534E variant has also been reported in another cohort in the U.S. [106] but not in the Middle Eastern or UK, or Chinese cohorts [102]. The *TTF1* germline mutation has been reported in two families with PTC and coexisting multinodular goiter. The inheritance was an autosomal dominant pattern. Transfection of the *TTF1* variant in rat normal thyroid cells overexpressed the gene resulting in increased cell proliferation, activated STAT3 and AKT signaling, and cyclin D2 expression. Because the data involving the association of *FOXE1, HABP2,* and *TTF1* with FNMTC is inconsistent among cohorts from various geographic locations, the validation in a larger cohort is required. Thus, the clinical testing of these genes in patients with FNMTC remains to be proven.

## 14. Proteomics in Thyroid Cancer

Major areas in molecular studies of various cancers include the use of proteomics for the following: (1) to provide a better understanding of the molecular pathogenesis leading to tumor development, progression, and metastasis and (2) to identify the biomarkers that improve diagnostic or prognostic accuracy. Recent advancements in molecular technology have enabled investigators to analyze proteins in various tissue types and body fluid using mass spectrometry in combination with various techniques to increase the proteome coverage. Two-dimensional gel electrophoresis is now replaced by liquid chromatography online coupled with mass spectrometry. The areas of improvement in thyroid cancer include the use of proteomics to aid preoperative diagnostic accuracy of a fine-needle aspiration biopsy in patients with an indeterminate thyroid nodule and the use of proteomics to identify the biomarkers, and therefore improve our understanding of thyroid cancer pathogenesis and guide management based on more accurate prognostication.

Because of different types of non-tumoral cells in thyroid tumor tissue that can mask the dysregulated protein expression from the tumor, biomarker discovery studies in thyroid remain challenging. Mass spectrometry imaging can overcome some of this limitation as it provides precise and localized information on the protein expression in the tissue [107]. The pattern of the proteome in fine-needle aspiration fluid of cPTC and TVPTC was compared to controls. There were 17 significantly upregulated protein spots in cPTC or TVPTC as compared with the controls. These included transthyretin precursor (TTR), ferritin light chain (FLC), proteasome activator complex subunit 1 and 2, alpha-1-antitrypsin precursor, glyceraldehyde-3-phosphate dehydrogenase (GAPDH), lactate dehydrogenase chain B (LDH-B), apolipoprotein A1 precursor (Apo-A1), annexin A1, DJ-1 protein, and cofilin-1. Twelve and three additional proteins were exclusively found in cPTC and TVPTC, respectively. The three proteins that were exclusively found in TVPTC (ferritin heavy chain, peroxiredoxin 1, and 6-phopohogluconaste dehydrogenase) were all involved in the oxidative stress response [108]. This novel finding provided insight into the PTC-related metabolic and oxidative stress response that could be useful in diagnostic classification and treatment. Another proteomic study by Ciergia et al. analyzed fine-needle aspiration (FNA) from patients with cPTC, TVPTC, and controls using two-dimensional gel electrophoresis and MS imaging and confirmed five upregulated proteins, previously described. These included L-lactate dehydrogenase B chain (LDHB), ferritin heavy chain, ferritin light chain, annexin A1 (ANXA1), and moesin. Of these, ANXA1 showed a sensitivity of 87% and a specificity of 94%. Using matrix-assisted laser desorption and ionization (MALDI) imaging mass spectrometry (IMS) and principal component analysis, the pattern of protein expression in various thyroid cancer pathology (hyperplastic nodules, Hurthle cell adenoma, cPTC, and medullary thyroid cancer) can distinguish cPTC from benign thyroid lesions and from that of medullary thyroid cancer [109]. A similar technique has been used in surgical frozen samples, and numerous proteins that were differentially expressed between thyroid cancer and the benign lesions were described with occasional overlapping results. Overexpression of galectin-1, galectin-3, and S100 proteins in PTC and FTC have been described in multiple studies [110,111,112]. Other proteins that were differentially expressed in thyroid cancer involve mitochondrial function, ROS, and oxidative stress response proteins, and proteins involved in protein folding (HSP40, HSP70, HSP90, calreticulin, and PDI A3) [113,114,115].

By compare PTC with and without lymph node metastasis in PTC with *BRAF* p.V600E, Park et al. showed increased vimentin in PTC with lymph node metastasis and upregulated HSP60 protein in PTC without lymph node metastasis [116]. Other proteins overexpressed in PTC with lymph node metastasis were S100-A6, S100-A10, and thioredoxin [117].

The role of liquid biopsy in thyroid cancer has been explored by studying various biomarkers in serum and urine. Although the concept of obtaining diagnostic or prognostic information from the easily accessible biospecimens that are noninvasive is attractive, the technical aspects related to sample collection, processing, storage, and analytical chemistry and selection methods for the candidate biomarkers can cause inconsistent results. Progress in proteomic studies has gradually minimized these biases. Using an optimized peptide extraction and matrix-assisted laser desorption/ionization time-of-flight (MALDI-TOF) mass spectrometry method, peptide signatures, mostly generated by exopeptidase activities and differential protease activity that are specific to the cancer type, can be identified in advanced prostate, bladder, and breast cancer [118]. Villanueva et al. optimized 12-peptide ion signature in thyroid cancer that had 95% sensitivity and 95% specificity for 19 correct predictions of 20 thyroid cancer and 20 controls. Ten of these peptides have been described as signature patterns in other solid cancers [119] Subsequent study by the same group, in 48 patients with metastatic thyroid cancer (and 48 controls), showed a high sensitivity (94%) and specificity (90%) in class prediction after protein quantification and multivariate analysis [120]. These peptides did not directly derive from tumor tissue but they represent differential activities of proteolytic events of the ex vivo coagulation and complement degradation pathway [118]. To identify differential protein expression in the serum of patients with iodine non-avid thyroid cancer and lung metastasis to those with iodine-avid metastasis, Song et al. identified and independently validated a downregulation of afamin, a highly glycosylated albumin with binding affinity to vitamin E which protects cells against oxidative damage [121]. Another technique called surface-enhanced laser desorption/ionization time-of-flight (SELDI-TOF) mass spectrometry has been used to analyze serum samples in patients with thyroid cancers. The sensitivity and specificity for distinguishing between PTC and normal or benign thyroid nodules ranged from 85.7% to 95.1% and 80% to 100%, respectively [122,123,124]. Haptoglobin alpha-1 chain was found to be upregulated in a PTC cohort from China and the level progressively increased with the clinical stages I to IV. Two additional proteins, apolipoprotein C-I, and C-III were downregulated in PTC and gradually decreased with the clinical stages I to IV [124]. In summary, the use of proteomic studies has been mainly for the identification of diagnostic and prognostic markers in thyroid cancer tumor tissue and in the serum of patients with thyroid cancer using various techniques involving mass spectrometry. With a limited sample size in each cohort, various proteome markers have been identified with a promising diagnostic and prognostic performance.

## 15. MicroRNAs in Thyroid Cancer

Because 95% of human transcriptome corresponds to non-protein coding RNAs, an increasing number of studies have suggested a role of ncRNAs in various biological processes. Several reports have demonstrated that ncRNAs expression pattern is associated with carcinogenesis. The ncRNAs are generally classified according to their sizes, i.e., a small ncRNAs group (20 to 30 nucleotides) that include microRNAs (miRNAs) and a long ncRNAs group (lncRNAs) that are greater than 200 nucleotides in length [125,126]. The miRNAs regulate gene expression at a transcriptional and post-transcriptional level and have been well described. In recent years, a growing number of studies aiming at elucidating the actions of lncRNAs have demonstrated their role in chromatin remodeling, chromatin interactions, and anti-sense function [127,128,129,130]. Expression alteration of ncRNA in cancer cells have suggested a role of ncRNA in the development of human malignancies.

Increased knowledge about the role ncRNAs in thyroid carcinogenesis has led to a significant improvement of our understanding of the molecular events involved in thyroid cancer initiation and progression. The roles of ncRNAs in thyroid cancer cell proliferation, dedifferentiation, metastasis, and immune evasion have been widely described. In this review, we will primarily focus on the well-described miRNAs and lncRNAs, their functions in thyroid carcinogenesis, and their use as diagnostic and prognostic markers.

MicroRNAs are single-stranded, 21 to 25 nucleotide long RNA sequence. Genes coding for miRNAs are transcribed into a primary miRNA (pri-miRNA) by RNA polymerase II in the nucleus. Pri-miRNA is then processed by Drosha in the nucleus into a precursor miRNA (pre-miRNA). Pre-miRNA is transported to the cytoplasm by exportin-5, where it is processed by Dicer, an RNAse III enzyme to release a mature miRNA. The role of miRNAs has been widely studied, and it is well accepted that miRNA bind to their mRNA target leading to mRNA degradation or repression of translation. A single miRNA can target several transcripts and conversely multiple miRNAs can alter the expression of the same mRNA. The miRNA expression pattern is tissue specific. The role of miRNAs has been demonstrated in many biological processes such as cell growth, death, differentiation, motility, and response to stress. The miRNA expression pattern in cancer cells has been the first indicator of their role in carcinogenesis. Several studies have revealed aberrant expression of miRNAs in cancer tissue as compared with normal cells. Functional studies performed in several human cancer models showed two distinct groups of miRNAs, oncomiRs and tumor suppressor miRNAs. OncomiRs are increased in cancer tissue which results in silencing tumor suppressors genes, and tumor suppressor miRNAs are downregulated in cancer cells and target oncogenes.

### 15.1. MicroRNAs in Differentiated Thyroid Cancer

Papillary thyroid cancer is the most common type of thyroid cancer; almost 80% of all thyroid cancer cases are PTC. Activation of the MAPK pathways driven by *BRAF* p.V600E mutation or RET/PTC translocation is the major oncogenic pathway regulating cell proliferation and carcinogenesis of PTC. Recent studies have discovered that the coexistence of *BRAF* p.V600E and TERT promoter mutations (C250T and C228T) in PTC was associated with increased recurrence, advanced stage, and shorter survival as compared with either mutation alone. Recently, the pan-genomic studies performed in a large cohort of patients with PTC suggested the key role of miRNAs in the transformation of normal follicular thyroid cells and thyroid cancer initiation. Furthermore, in-depth genomic analysis and functional studies uncovered the fundamental functions of several miRNAs in PTC.

The aberrant expression of miR-146 has been found in several cancers, and analysis of the peripheral blood of 128 patients showed high expression of both miRNAs in patients with PTC as compared with patients with benign lesions and healthy controls [131]. Moreover, analysis of the association of miR-146a and miR-146b with the clinical and pathological features of thyroid cancer showed a higher expression in patients with the more aggressive disease [131]. In silico analysis and functional studies have shown that miR-146 by targeting alpha 2,8-sialyltransferase (*ST8SIA4*) and the retinoic *RARB* is implicated in thyroid carcinogenesis [132,133]. *ST8SIA4* is involved in various biological processes including cell adhesion and metastasis partially by regulating the PI3K/Akt pathway in follicular thyroid cancer cells. *RARB* codes for the retinoic acid receptor beta (RARβ) which binds to the retinoic acid, the active form of Vitamin A that plays a major role in cell proliferation and differentiation. In thyroid cancer, retinoic acid treatment has been used to redifferentiate the follicular cells, increase NIS expression, and improve radioactive iodine uptake [134,135,136]. Thus, reduction of RARβ expression in thyroid cancer is associated with cell proliferation and dedifferentiation and is a major limitation for radioactive iodine therapy. Comparison of miRNAs expression pattern in serum samples from patients with radioactive-iodine avid metastasis lesions versus non-avid demonstrated upregulation of miR-106a in the non-avid group and that miR-106 directly targets *RARB* [137]. Taken together, these findings suggest that targeting miR-146 and miR-106a might be a new therapeutic strategy to overcome thyroid cancer dedifferentiation and increase the iodine uptake in cancer cells.

The miR let-7 family play important roles in many biological processes and have been associated with cell transformation and cancer initiation. The miR let-7 family promotes differentiation and function as a tumor suppressor in several human cancers [138,139,140]. A recent study, exploring the role of the miR let-7 family in thyroid carcinogenesis and demonstrated the tumor suppressor function of miR let-7e by targeting HMGB1. HMGB1 is overexpressed in several human malignancies and plays a central role in carcinogenesis through a different mechanism. On the one hand, for example, secreted HMGB1 binds to various receptors to activate oncogenic pathways such as the NFkB pathway and the PI3K pathway. On the other hand, HMGB1 can trigger autophagy, a mechanism used by cancer cells to promote cell proliferation and drug resistance [141].

Analysis of The Cancer Genome Atlas and Gene Expression Omnibus demonstrated a significant reduction of miR-486-5p in PTC as compared with normal thyroid tissue. Analysis of the clinical and pathological features of patients with thyroid cancer showed a significant association of low miR-486-5p with advanced overall stage, lymph nodes metastasis, distant metastasis, and recurrence. In addition, patients with low miR-486-5p showed a shorter overall survival as compared patients with high miR-486-5p level. These data suggest that miR-486-5p is implicated in thyroid cancer initiation and progression [142]. *FBN1* was identified as a direct target of miR-486-5p in PTC cells [143]. *FBN1* is upregulated PTC and implicated in cell growth, motility, and epithelial-mesenchymal transition [143,144].

The role of miR-7-5p has been widely studied in various cancers, analysis of its expression profile in thyroid cancer showed a significant reduction of miR-7-5p expression in PTC tissue as compared with adjacent normal tissue. Analysis of the biological function of miR-7 in thyroid cancer demonstrated the role of miR-7 in thyroid cell growth and metastasis by targeting cyclin-dependent kinase subunit 2 (*CKS2*) and p21-activated kinase 1 (*PAK1*). CKS2 and PAK1 are highly expressed in PTC and implicated in cell cycle regulation, apoptosis, and metastasis [145,146]. Moreover, a recent study demonstrated a direct interaction between TERT and miR-7-5p. Silencing of miR-7-5p increased *TERT* expression in thyroid cancer cells. These findings are of interest in thyroid cancer as high *TERT* expression has been associated with aggressive behavior of thyroid tumors and a higher risk of recurrence [147].

In several human malignancies, miR-221 and miR-222 have been identified and miR-222 and miR-221 have been found overexpressed in tissue and serum from patients with thyroid cancer, associated with a higher risk of recurrence and aggressive clinical and pathological features of the tumor [148,149,150]. A recent study suggested that miR-222 mediates its effects on the epithelial-mesenchymal transition and metastasis by targeting protein phosphatase 2 regulatory subunit B alpha (*PPP2R2A*). PP2R2A is a master regulator of the cell cycle that inactivates the PI3K/Akt pathway by inhibiting the phosphorylation of Akt in several cancers including thyroid cancer [151,152,153]. In vitro and in vivo studies performed using TPC1 cell lines identified *TIMP3* as a direct target of miR-221 [154]. *TIMP3* is a dual inhibitor of MMPs and ADAM in the tumor microenvironment, and the lack of *TIMP3* has been associated with angiogenesis and cancer cell invasion. Thus, accumulating evidence implicates miR-221 and miR-222 not only in cancer initiation but also in cancer progression, suggesting that miR-221 and miR-222 could serve as a prognostic marker for aggressive thyroid tumors.

The miR-145 is a downregulated tumor suppressor miRNA in thyroid cancer. Functional studies and preclinical studies identified *Akt*3 and the dual-specificity phosphatase 6 (*DUSP6*) as direct targets of miR-145 [155,156]. Akt is a key protein of the PI3K/Akt signal transduction cascade that plays a central role in cell proliferation, apoptosis, and motility in thyroid cancer [157]. DUSP6 is a member of the MAPK phosphatases family, is highly expressed in PTC, and plays a pro-oncogenic role in thyroid tumorigenesis by regulating the ERK1/2 pathway, enhancing cell proliferation, and invasiveness [158]. Studies investigating the mechanism responsible for the aberrant expression of miR-145 in thyroid cancer, demonstrated the role of two ncRNAs in suppressing miR-145 expression, TUG1, and CircNUP214 [159,160].

### 15.2. MicroRNAs in Poorly Differentiated and Anaplastic Thyroid Cancer

Many miRNAs show similar expression pattern in differentiated and undifferentiated thyroid cancers, however, miR-200 and miR-30 families are exclusively dysregulated in anaplastic thyroid cancer, suggesting a role of these miRNAs in cancer progression and metastasis.

The miR-200 family is described as a tumor suppressor in many cancers. The role of miR-200s in the modulation of the EMT has been demonstrated in several studies by targeting ZEB1, ZEB2, SNAI2, and TGF-β2 [161,162,163]. A decrease in miR-200 expression is associated with cancer cells dedifferentiation, invasion, migration, and metastasis. Analysis of the expression profile of miR-200s in thyroid cancer showed that ATC and PDTC harbor the lowest expression of miR-200a, miR-200b, and miR-200c as compared with WDTC and normal thyroid tissue [164]. Analysis of the molecular mechanisms modulating miR-200 expression in metastatic thyroid tumors demonstrated the role of EGF/EGFR signaling in the repression of miR-200s through the activation of Rho/ROCK and the TGB-β pathway, and thereby enhancing EMT transcription factor expression and promoting tumor invasiveness [165,166,167]. In agreement with these findings, inhibition of TGFBR1 increased miR-200 expression [164]. These findings suggest that miR-200 is a marker for cell differentiation, and the loss of miR-200s is a promising predictive marker for metastatic tumor.

The miR-30 family members have been implicated in several biological processes in cancer cells by functioning as tumor suppressors in multiples cancer models [168,169,170]. Analysis of miR-30a family members expression in thyroid cancer tissues revealed a significantly downregulated expression in ATC and PDTC as compared with well-differentiated tumors and normal thyroid tissue and was associated with a shorter survival [164,171]. The observed altered expression of miR-30s suggests a function in EMT and cancer metastasis. Ectopic expression of miR-30s was associated with MET-like phenotype reduced Vimentin and *ZEB2*, two markers of invasive phenotype of thyroid cancer [164]. These observations suggest an anti-metastatic role of the miR-30 family in ATC. In addition, a study done by our group demonstrated a critical role of miR-30a in thyroid cancer progression by targeting lysyl oxidase expression (LOX). LOX is an amine oxidase which has been implicated in cancer invasion, adhesion, and ECM modulation through its intracellular and extracellular functions. Our findings showed a significant increase of LOX in ATC and demonstrated its role in ATC cell growth, invasion, and metastasis in vitro and in vivo [171]. Collectively these findings suggest the miR-30 family as a new diagnostic and prognostic marker for metastatic thyroid cancer. Despite the numerous studies describing the role of miR-30 family members in cancer progression, the molecular mechanism responsible for the altered expression of these miRNAs is not fully understood. A recent study revealed that the oncogenic lncRNA CNALPTC1 sponges miR-30s, thus downregulating miR-30 expression and enhancing EMT markers such as SNAI1 and vimentin expression.

The miR-17-92 was found overexpressed in ATC. Functional studies have demonstrated the oncogenic role of miR-17-92 cluster in ATC-derived cells; miR-17-92 by targeting tumor suppressor genes, such as *PTEN*, promote tumor growth and inhibit apoptosis in ATC. Furthermore, induction of *BRAF* V600E mutation increased miR-17-92 cluster expression in thyroid cancer cells [172].

Anaplastic thyroid cancer is caused by accumulated genetic changes that have been implicated in drug resistance and the lack of response to targeted therapies. Currently, it is proposed that aberrant expression of miRNAs in cancer cells could play a role in drug resistance by modulating the expression of target genes involved in response to the treatment. For example, miR-30d, by targeting Beclin 1, negatively regulates autophagy, and promotes apoptosis in cisplatin-treated ATC-derived cells [173]. In addition, miR-27-3b has also been associated with ATC drug resistance, for example, miR-27-3b, by targeting PPARγ in ATC, promotes ATC cell resistance to doxurubicin [174].

Resistance to BRAF inhibitors is an active area of research, and ATC patients often develop resistance to the agents targeting *BRAF* V600E or the MAPK pathway. The microRNAs, miR-125a, miR-204, and miR-211 have been proposed as key players in the acquired resistance to BRAF inhibitor in melanoma, highlighting the clinical relevance of miRNA in cancer therapy [175]. However, no similar studies have been done in metastatic thyroid tumors.

### 15.3. Prognostic and Diagnostic Value of miRNA in Thyroid Cancer

Fine-needle aspiration biopsy (FNAB) is the standard of care to investigate thyroid nodules. Reporting Thyroid Cytology characterizes FNA samples into the following six categories with increasing risk of malignancy: I, nondiagnostic (5% to 10% risk of malignancy); II, benign (<3% risk of malignancy); III, atypia of undetermined significance or follicular lesion of undetermined significance (AUS/FLUS) (10% to 30% risk of malignancy); IV, follicular neoplasm or suspicious for a follicular neoplasm (FN/SFN) (up to 35% risk of malignancy); V, suspicious for malignancy (50% to 75% risk of malignancy); and VI, malignant (97% to 99% risk of malignancy) [176,177]. Although FNA biopsies allow an accurate preoperative classification of the thyroid nodules, about 30% of FNA samples are cytologically indeterminate for malignancy, which makes the clinical management very challenging. To reduce the unnecessary surgeries, as often cytologically indeterminate nodules are benign lesions, the use of molecular markers in FNA samples has emerged [178]. Molecular markers have been tested in FNA samples to improve the accuracy of the diagnosis of malignant nodules.

To improve the diagnostic accuracy, a study performed in a large number of FNA samples combined miRNA, DNA, and mRNA analyses. The miRNA classifier was generated using 240 surgically resected benign or malignant thyroid nodules and validated in 54 surgically removed nodules and 235 FNA samples. The classifier of 10 miRNAs included the following: miR-29-b-1-5, miR-31-5p, miR-138-13p, miR-139-5p, mir-146b-5p, mir-155, miR-204-5p, miR-222-3p, miR-375, and miR-551b-3p and identified 64% of malignant cases and 98% of benign cases. When combined with mutation detections, the miRNA expression classifier showed a sensitivity of 89% and specificity of 85%. These findings suggest that combining mutation detection and miRNA classifier in indeterminate FNA offer valuable information for the diagnostic of thyroid nodules, thus offering more personalized treatments to the patient [179].

A comparison of the expression profile of miRNA in FA and FTC in fresh frozen tumors showed a small number of miRNAs differentially expressed in cancer tissues as compared with benign lesions. Dysregulated miRNAs were used to generate a classifier tested in 44 FNA samples negative for known somatic mutations, among which 33 were classified as indeterminate. The data analysis identified two miRNAs classifiers, miR-484/miR-148b-3p with a sensitivity of 89% and a specificity of 87% and miR-484/miR-139-5p classifier with a sensitivity of 64% and a specificity of 100% [180].

Analysis performed in 120 FNA with indeterminate cytology combining *BRAF* p.V600E mutation status, miR-221, miR-222, and galectin-3 expression showed a sensitivity of 73.5%, a specificity of 89.8%, and a diagnostic accuracy of 75.7%, a positive predictive value of 80.6%, and a negative predictive value of 85.5% [181].

Another recent study done in indeterminate FNA samples from patients who had surgery developed a new miRNAs classifier of 11 miRNAs that was trained in 78 FNA samples and validated in a cohort of 95 FNA smear slides. In the training set, the miR-THYpre test reached 89.7% sensitivity, 92.3% specificity, 90% negative predictive value and 92.1% positive predictive value. In the validation cohort, the miR-THYpre reached 94.6% sensitivity, 81% specificity, 95.9% negative predictive value, and 76.1% positive predictive value [182].

Taken together, all these findings suggest that miRNAs classifiers can be considered for preoperative use for indeterminate thyroid nodules to reduce the number of unnecessary surgeries.

## 16. Metabolic Reprogramming in Thyroid Cancer

Metabolomics involves the measurements of the metabolites in cells. The increasing number of studies has described metabolic alterations in cancer cells. Understanding the metabolic changes occurring in cancer cells will lead to new approaches for cancer therapy targeting tumor metabolism and the discovery of new predictive markers. Recent advances in cancer research have provided new insight into the connection between genetic alteration-mediated signaling pathways and cancer cell metabolism. Cancer cell metabolism reprogramming is also involved in the malignant transformation by regulating the pro-survival pathways that leads to sustained cell growth, to cancer progression and drug resistance [183,184,185]. *BRAF* and *KRAS* mutations have been associated with increased in glucose uptake, lactate production and phosphoserine biosynthesis suggesting a shift toward glycolysis pathway in colorectal cancer [186]. Furthermore, a study done in melanoma cells showed that *BRAF* activating mutation was associated with reduced TCA cycle and oxidative phosphorylation and increased glycolysis as a source of energy [187] and *BRAF* p.V600E inhibitor-resistant cells switch to glutaminolysis to sustain their survival rate [188]. Although *BRAF* p.V600E is the most common mutation in thyroid cancer, the association between activating mutations and the metabolic reprogramming has not been clearly demonstrated. A recent study, showed in *BRAF* p.V600E mutated cell lines, that the mutation is associated with increased HIF-1α expression which in turn represses PGC-1β expression. PGC-1β regulates mitochondrial biogenesis and oxidative phosphorylation (OXPHOS). Moreover, MEK inhibitor increased OXPHOS-related genes in BRAF p.V600E mutated cell lines [189]. These findings suggest that oncogenic activation of *BRAF* inhibits OXPHOS in mitochondria and promotes aerobic glycolysis to sustain cancer cell growth.

The analysis of the metabolome in thyroid cancer tissues showed an elevated level of Lactate in the malignant tumors compared to the adjacent normal tissue. increased lactate is often associated with high glucose metabolism [190].

Another study, analyzing of the metabolic profiling in PTC tissues showed that the metabolites of the galactose metabolic pathway (mannose, sorbitol, galactinol, and glucose) are significantly altered in thyroid cancer tissue compared to normal thyroid. These findings suggest that the inactivation of alpha-galactosidase (GLA), a key enzyme of the galactose metabolism might be responsible for the lower levels of these metabolites in thyroid cancer [191]. A study analyzing the metabolic profiling in FFPE samples from patients with FTC, FA or FV-PTC, identified 28 metabolites significantly different between the three types of histology. These metabolites were associated with lipids metabolism, mitochondrial electron transport chain, galactose and pentose phosphate metabolism, gluconeogenesis, and glutamate metabolism. Taken together, these findings suggest a higher energy needs in cancer cells compared to normal thyroid cells [192].

## 17. Epigenetic Changes in Thyroid Cancer

The role of epigenetic alterations in thyroid cancer initiation has been extensively studied. Several studies have identified a large panel of genes involved in cell proliferation, apoptosis, cell cycle regulation, invasion and migration that are epigenetically modified thus leading to genetic alterations associated with cancer initiation and progression. Analysis of the methylation pattern of thyroid cancer has shown an association between the methylation signature, the histology, and the mutation status. PTC is characterized by more hypomethylation than hypermethylation compared to normal thyroid tissue whereas FTC harbor more hypermethylation than hypomethylation compared to normal tissue. Among PTC, FV-PTC exhibits only few aberrant changes with a methylation pattern not very different from normal follicular cells. ATC and PDTC carry a higher number of methylation changes among all thyroid cancers with more hypomethylation than hypermethylation. The effect of methylation alterations in thyroid carcinogenesis is demonstrated by downregulation of several tumor suppressors thyroid cancer cells, such as; *TSHR*, *PTEN, RASSF1A, CDKN2A, DAPK1, TIMP3, ECAD, and RAP1GAP* [193,194,195,196,197,198]. On the other hand*, MAP17, CXCL12, HORMAD2, and PFKFB2* are hypomethylated and upregulated in aggressive thyroid cancer [199,200,201,202].

Studies analyzing the association between the mutations status of the tumor and the methylation pattern revealed that *BRAF* p.V600E, the most common mutation in PTC is associated with increased global hypomethylations whereas *RAS* mutated tumors have more hypermethylated loci [22,203,204]. Taken together, these findings suggest that oncogenic activation have a significant impact on gene expression by modifying their methylation status.

In addition to methylation, post-translational modifications of histones regulate gene expressions by modifying the chromatin structure. Thus, regulating major cellular processes such as cell cycle, cell death, and cell metabolism [205]. However, the role of histones modification in thyroid cancer initiation and progression is not well studied. Increased acetylated histones in NIS promoter inhibits its expression. Mechanistic studies showed that the treatment of thyroid cancer cells with HDAC inhibitors restores NIS expression and increase iodine uptake in cancer cells [206,207]. The thyroid transcription factor TTF-1 is silenced in aggressive thyroid cancer that is associated with a loss NIS expression and iodine uptake. Analysis of the epigenetic events involved in the silencing of TTF1 revealed that DNA hypermethylation, increased dimethyl-H3-lys9 and reduced acetyl-H3-k9 are involved in the loss of TTF1 [208]. Thus, a DNA demethylating agent could have a clinical benefit in thyroid tumors with reduced or lost TTF1 expression.

## 18. Conclusions

In summary, the advances in molecular biotechnology with the availability of high-throughput assays in multi-omic platforms with the integrated analyses have improved our understanding of the molecular abnormalities in thyroid cancer initiation and progression. Many of the findings can be further applied to the clinical use to improve diagnostic and prognostic accuracy and to improve treatment efficacy.
